# Hematopoietic Stem and Progenitor Cells Can Be Enriched by Implanting Biomaterial into Spatium Intermusculare

**DOI:** 10.1155/2015/398642

**Published:** 2015-01-28

**Authors:** Jia-Bei Tong, Xiao-Yun Wu, Ge-Liuchang Jia, Kui-Jun Zhao, Shi-Li Wang, Zhi-Jie Ma

**Affiliations:** ^1^Department of Pharmacy, Beijing Friendship Hospital, Capital Medical University, No. 95, Yongan Road, Xicheng District, Beijing 100050, China; ^2^Key Laboratory for Biotech-Drugs Ministry of Health, Key Laboratory for Modern Medicine and Technology of Shandong Province, Key Laboratory for Rare & Uncommon Diseases of Shandong Province, Key Laboratory for Virology of Shandong Province, Shandong Medicinal Biotechnology Centre, Shandong Academy of Medical Sciences, 18877 Jingshi Road, Jinan 250000, China; ^3^Department of Technology, Beijing Jingmeng Stem Cell Technology Co., Ltd., 5-2 Shangdi East Road, Beijing 100085, China

## Abstract

Hematopoietic stem and progenitor cells (HSPCs) have been used successfully to treat patients with cancer and disorders of the blood and immune systems. In this study, we tried to enrich HSPCs by implanting biomaterials into the spatium intermusculare of mice hind limbs. Gelatine sponges were implanted into the spatium intermusculare of mice and then retrieved after 12 days. The presence of HSPCs in the migrating cells (MCs) was detected by phenotypically probing with CD34^+^Sca-1^+^ and functionally confirming the presence of using colony-forming cell assay and assessing the long-term reconstitution ability. The frequency of CD34^+^, Sca-1^+^, and CD34^+^Sca-1^+^ cells and colony formation unit in the MCs was much higher than that in the bone marrow (BM). Moreover, transplanted MCs were able to home to BM, muscle, and spleen, which induced an efficient long-term hematopoietic reconstitution in vivo. In addition, HSPCs within the MCs originated from the BM. Furthermore, the administration of G-CSF greatly reduced the time of implantation, and increased the number of MCs and frequency of HSPCs in the MCs. These data provide compelling evidence that HSPCs can be enriched by implanting biomaterial into spatium intermusculare. Implantation of biomaterial may be seen as the first step to a proof of their applicability to clinical practice in enriching HSPCs.

## 1. Introduction 

Hematopoietic stem and progenitor cells (HSPCs) are arguably the most well-characterized tissue-specific stem cells, with decades of basic research and clinical application providing not only a profound understanding of the principles of stem cell biology, but also its potential pitfalls. It is our belief that emerging stem cell fields can benefit greatly from an understanding of the lessons learned from the study of HSPCs. HSPCs transplantation is an increasingly accepted effective treatment option for patients with severe autoimmune diseases refractory to conventional treatment [1] and has been used successfully in patients with multiple sclerosis [2], rheumatoid arthritis [3], systemic sclerosis [4], systemic lupus erythematosus [5], and Crohn's disease [6, 7]. Currently, HSPCs are mainly collected from either PB or BM for basic research and clinical application. BM is the dominant organ containing HSPCs during adulthood. Moreover, a small fraction of HSPCs constantly circulate between BM and PB without any stimulation [8]. So HSPCs can be collected from PB via mobilization and apheresis. In principle, on the way from PB back to the BM, HSPCs can settle down if the proper environment is provided. Supporting this concept, several extramedullary tissues are identified as HSPC-containing organs, including the liver [9], spleen [10], muscle [11], adipose tissue [12], and thoracic duct [13]. The mechanism underlying HSPCs maintenance in these extramedullary organs remains unclear, though the HSPCs still conserve their function properly. Adipose tissues, one of the most abundant tissues in the human body, can be easily obtained from patients using conventional liposuction methods and removed without functional defect [14, 15]. However, the frequency of HSPCs in the adipose tissues is much lower than that in the BM, despite containing HSPCs. According to some reports, the estimated amount of HSPCs in total adipose tissues would be equivalent to approximately 0.2% of that of HSPCs in the total BM [12, 16, 17].

In our previous study, we successfully implanted sponges into the spatium intermusculare of mice hind limbs and enriched MSCs in MCs after 12 d. We speculated that this might be due to absorption of gelatin sponge and cell migration and proliferation effects [18]. Our previous study also demonstrated that cells captured from spatium intermusculare by porous material could be differentiated into hematopoietic cells in vitro [19]. In addition, we elucidated that MSCs in the MCs originated from the PB by BM transplantation. The MSCs in the BM could move from the BM to systemic circulation, home to the muscle, and migrate into biomaterials [18]. Besides MSCs, HSPCs exist in the PB and BM [20]. Moreover, HSPCs also show migration capabilities. Based on these facts, we hypothesize that HSPCs in vivo like MSCs could migrate into biomaterials when biomaterials are implanted into the spatium intermusculare.

To address our hypothesis, in this study, we implant biomaterials into the spatium intermusculare of mice hind limbs and characterize presumable HSPCs in MCs by phenotypic and functional analyses in vitro and in vivo. We also elucidate the origin of HSPCs in the MCs. Finally, we determine whether the number and frequency of HSPCs in MCs could be increased by G-CSF administration.

## 2. Materials and Methods

### 2.1. Mice

C57BL/6 mice, 6–8 weeks of age, were obtained from the Laboratory Animal Center of Shandong University (Jinan, China) and housed in microisolator cages in a temperature- and humidity-controlled environment. Mice were fed Purina Lab Chow and water* ad libitum*. The experimental animal protocol was approved by the University of Shandong IACUC committee.

### 2.2. Preparation of Materials

Gelatin sponges (Gelfoam) were purchased from Jinling Company (Nanjing, China) and prepared as described previously [18, 19]. Briefly, sponges were processed into the wafer with radius of 2.5 mm and thickness 5 mm and then sterilized by ethylene oxide. All operations were conducted in a biological safety cabinet.

### 2.3. Surgical Procedure

Gelatin sponges were implanted into mice as previously described [18, 19]. Briefly, sponge pieces were soaked in PBS, pH7.2. Mouse was anesthetized with diethyl ether and the medial thigh was shaved and swabbed with alcohol. An approximately 10 mm cut was made in the skin of each mouse's medial thigh and the deep fascia was minimally cut with sterile scissors so that the muscle group could be exposed. Two sponge pieces per mouse were gently inserted into the spatium intermusculare of the exposed muscle group, taking care that the sponge pieces were under the subcutaneous fascia. The wound was closed with wound clips.

### 2.4. Collection of BM and MCs

At the 12th day after implanting, mice were killed by cervical dislocation, the sponges and femurs were taken out, and then MCs and BM cells were immediately collected as follows.

BM aspirates were collected by flushing the femur with sterile PBS with 2% FBS (Gibco). Cells were washed once and resuspended in 10 mL of growth medium (DMEM containing 20% FBS, 1% penicillin/streptomycin (100 units/mL), 1% L-glutamine, and 2.5 *μ*g/mL amphotericin (all from Invitrogen)). Cell numbers were counted with a hemocytometer.

MCs were collected by shredding the sponges with forceps and squeezing the sponge pieces in sterile PBS with 2% FBS. Sponge debris was permitted to settle before the cell-containing supernatant fluid was removed. Cells were washed once and resuspended in 10 mL of growth medium. Cell numbers were counted with a hemocytometer.

BM cells and MCs were used for flow cytometric analysis, CFU assay, and long-term reconstitution transplantation.

### 2.5. Flow Cytometry Assay

MCs and BM cells were analyzed to determine the proportion of CD34^+^, Sca-1^+^, and CD34^+^Sca-1^+^cells. To detect CD34^+^, Sca-1^+^, and CD34^+^Sca-1^+^ cells in the MCs and BM cells, isolated MCs and BM cells were resuspended in 2% FBS containing PBS and stained with FITC-conjugated rat anti-mouse Sca-1 and PE-conjugated rat anti-mouse CD34 (all from Biolegend). Dead cells were excluded by 7-aminoactinomycin D (7-AAD; Invitrogen). Flow cytometric analyses for the stained cells were performed using the LSRII instrument (Becton Dickinson).

### 2.6. Immunofluorescence Staining

MCs and BM cells were fixed with 1% paraformaldehyde in PBS for 10 min and blocked with 5% goat serum in PBST (0.3% Triton X-100 in PBS) for 10 min at room temperature. The cells were incubated with PE-conjugated rat anti-CD34 and FITC-conjugated rat anti-Sca-1. After several PBST washes, cells were visualized by fluoroscopy. The percentage of positive cells was calculated by counting positively stained cells in the randomly chosen fields.

### 2.7. In Vitro CFU Assay

MCs and BM cells were suspended in 2% FBS containing DMEM and then mixed with methylcellulose-based semisolid medium (M3434; StemCell Technologies) by vortexing. We plated 6 × 10^4^ MCs and 3 × 10^5^ BM cells per 1 mL media mixture on a 35 mm petri dish (BD Falcon), which was cultured in 5% CO_2_ humidified incubator at 37°C. The numbers of colonies were counted and types of colonies classified under inverted microscope (Ziess) by morphologic criteria 14 days after plating.

### 2.8. Long-Term Reconstitution Assay

MCs and BM cells derived from male mice were collected by the method described above and counted. Forty-five female C57BL/c mice weighing 18–22 g were irradiated with 8.5 Gy and then randomly divided into 3 groups with 15 mice each. Group 1 was injected on day 2 after irradiation with 0.2 mL of PBS, group 2 was injected on day 2 after irradiation with 0.2 mL of MCs (1.0 × 10^7^ cells), and group 3 was injected on day 2 after irradiation with 0.2 mL of BM cells (1.0 × 10^7^ cells). The female recipients from each group were killed by cervical dislocation 16 weeks after transplantation and DNA samples were extracted, respectively, from the BM, muscle, and spleen. Male-derived cells were evaluated by PCR. The long-term reconstitution capacity of HSPCs in the MCs was further analyzed by CFU assay after 16 weeks of transplantation; BM cells of all recipients from 3 groups were cultured on methylcellulose-based medium at 37°C for 2 weeks. The types of colonies were classified under inverted microscope (Ziess) by morphologic criteria; individual colonies were plucked and further evaluated by Y-chromosome PCR.

### 2.9. BM Transplantation

To determine the origin of HSPCs in the MCs, we performed BM transplantation. BM cells were obtained from male mice and transplanted into female mice, and then sponges were implanted into the spatium intermusculare of female recipients at 24 hours after transplantation. The sponges were retrieved from recipients at 12 days after implantation and MCs were further analyzed, BM was obtained from female recipients as control. MCs and BM cells were cultured on methylcellulose-based medium at 37°C for 2 weeks. The individual colonies were plucked and were evaluated by Y-chromosome PCR.

### 2.10. Y-Chromosome PCR Analysis

PCR was performed to determine if any male-derived cells were present. Individual hematopoietic colonies were plucked and resuspended in 10 mL of PBS and treated with 15 mL of proteinase K buffer (2 M KCl, 1 M Tris HCl, 1 M MgCl_2_, 20 mg/mL gelatin, 1% NP40, 1% Tween 20, and 100 *μ*g/mL Proteinase K) for 3 h at 56°C. Samples were denatured by heating at 95°C for 10 min and then used in Y-chromosome PCR reactions. Mouse Y-chromosome specific Sry primers: (sense primer) 5′-ATTTATGGTG TGGTCCCG-3′; (antisense primer) 5′-GCTGTAAAATGCCACTCC-3′. PCR was initiated at 94°C for 4 minutes, followed by 30 cycles of 94°C for 1 minute, 62°C for 1 minute, and 72°C for 2 minutes, followed by a final cycle of 72°C for 5 minutes. To perform PCR analysis in BM, muscle, and spleen, processed DNA samples were amplified as described above. All PCR amplifications included a male (positive) and female (negative) control. The resulting products were analyzed by electrophoresis on a 1.0% agarose gel and stained with ethidium bromide. The expected size of amplified DNA fragment was 239 base pairs.

All experiments were run in triplicate.

### 2.11. G-CSF Administration

Sponges were prepared by immersion in 50 ng/mL G-CSF and then implanted into the spatium intermusculare of mice hind limbs. Thirty mice were killed on successive six days (5 mice each day) after implanting. The sponges were retrieved and weighed, and then MCs were collected and counted with a hemocytometer. MCs were further used for flow cytometric analysis and CFU assay.

### 2.12. Statistics

Values presented are means plus or minus SD. Significant differences between means were determined by Student's *t*-test or analysis of variance followed by the Student-Newman-Keuls test. Significance was set at a value of *P* less than 0.05.

## 3. Results 

### 3.1. The MCs Contained Phenotypic HSPCs

**Figure 1:**
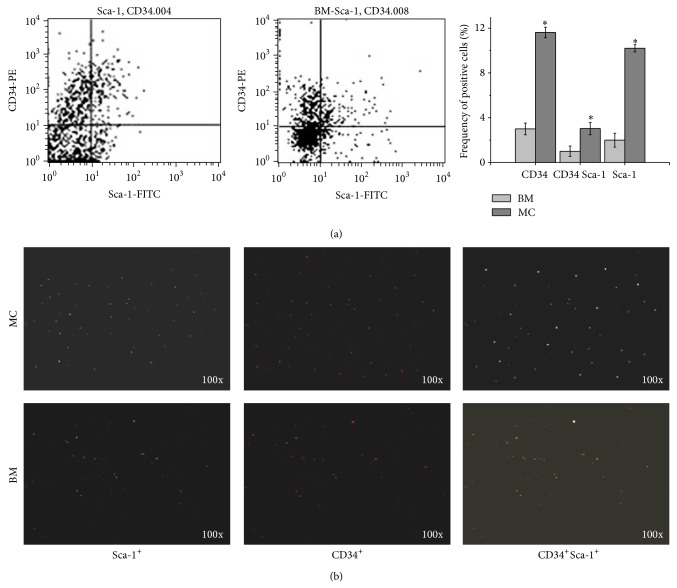
The migrating cells (MCs) contained phenotypic hematopoietic stem and progenitor cells (HSPCs). (a) Representative flow cytometric profiles of CD34^+^, Sca-1^+^, and CD34^+^Sca-1^+^ cells in the MCs and bone marrow (BM). MCs and BM were pregated for live cells and then were analyzed for CD34^+^, Sca-1^+^, and CD34^+^Sca-1^+^ expression. The percentages of CD34^+^, Sca-1^+^, and CD34^+^Sca-1^+^cells in the total MCs (*n* = 5) or BM (*n* = 5) were expressed as mean ± SD; ^*^ *P* < 0.05 versus BM. (b) Immunofluorescence staining for CD34^+^ (red), Sca-1^+^(green), and CD34^+^Sca-1^+^ (yellow) cells in the MCs and BM. Original magnifications ×100.

To determine whether the MCs contained phenotypic HSPCs, flow cytometric analysis was performed to detect CD34^+^, Sca-1^+^, and CD34^+^Sca-1^+^ cells. The frequency of CD34^+^, Sca-1^+^, and CD34^+^Sca-1^+^ cells was 11.62% ± 0.47%, 10.21% ± 0.32%, and 3.03% ± 0.55% in the MCs (*n* = 5), respectively, and it was 5.69% ± 0.52%, 2.50% ± 0.65%, and 1.89% ± 0.47% in the BM (*n* = 5). Obviously, HSPCs were present in higher proportions in MCs than those in BM (all *P* < 0.05) (Figure 1(a)). We further identified the CD34^+^, Sca-1^+^, and CD34^+^Sca-1^+^ cells in the MCs using immunofluorescence staining (Figure 1(b)). The frequency of CD34^+^, Sca-1^+^, and CD34^+^Sca-1^+^ cells in the MCs was significantly higher than that in the BM. These results provided compelling evidence that the MCs contained phenotypic HSPCs, and their frequency was significantly higher than that in the BM.

### 3.2. The MCs Contained HSPCs Capable of Forming Hematopoietic Colonies

**Figure 2:**
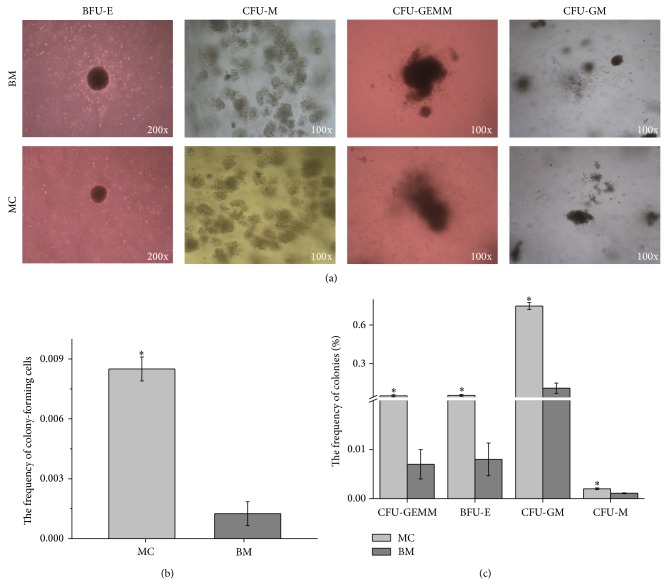
The migrating cells (MCs) contained functional hematopoietic stem and progenitor cells (HSPCs). (a) Colony-forming unit (CFU) assay of MCs and bone marrow (BM) cells. The colonies were classified by morphologic criteria; representative colonies were shown. Burst-forming unit-erythroid: BFU-E, colony-forming-unit-macrophage: CFU-M, colony-forming-unit-granulocyte, macrophage: GFU-GM, and colony-forming-unit-granulocyte, erythroid, macrophage, megakaryocyte: CFU-GEMM. (b) The frequency of colonies was shown; values were normalized to the number of input cells. The bars represented mean ± SD (*n* = 5); ^*^ *P* < 0.05 versus BM. (c) The frequency of each colony type (BFU-E, GFU-GM, CFU-M, and CFU-GEMM) in MCs was shown; values were normalized to the number of input cells. The bars represented mean ± SD (*n* = 5); ^*^ *P* < 0.05 versus BM.

To test whether the MCs had functional hematopoietic activity in vitro, the CFU assay was performed by plating freshly isolated MCs on methylcellulose-based medium. Morphologic criteria analyses demonstrated that the MCs gave rise to various types of colonies, such as BFU-E, CFU-M, CFU-GM, and CFU-GEMM. The size and appearance of each type of colony derived from the MCs were identical to the colonies derived from the BM (Figure 2(a)). The frequency of colonies in MCs was higher than that in BM (Figure 2(b)). The approximation ratio of hematopoietic colonies (MCs : BM) was 4–6-fold. Moreover, the frequency of each colony type (BFU-E, GFU-GM, CFU-M, and CFU-GEMM) in MCs was proportionally higher than that in BM (Figure 2(c)), but the proportion (BFU-E : GFU-GM : CFU-GEMM = 10.3% : 78.8% : 10.9%) of each colony type in MCs was similar to that in BM (11.2% : 80.0% : 8.8%), indicating that MCs possessed functional HSPCs that could give rise to several types of blood cells, and the frequency of HSPCs in the MCs was much higher than that in the BM.

### 3.3. HSPCs in the MCs Were Capable of Long-Term Hematopoietic Reconstitution In Vivo

**Figure 3:**
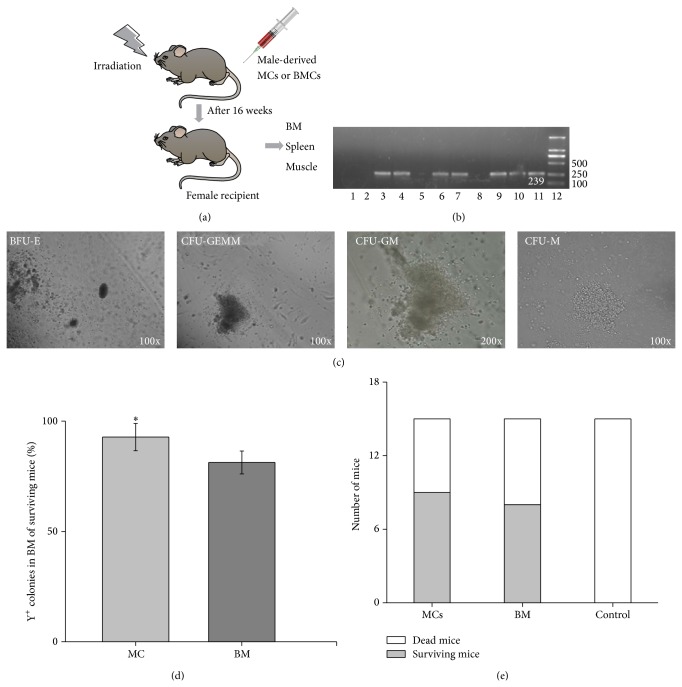
Hematopoietic stem and progenitor cells (HSPCs) in the migrating cells (MCs) were capable of long-term reconstitution in hematopoiesis in recipient mice. (a) Experimental scheme. After irradiation, the mice underwent transplantation with PBS (control), 1 × 10^7^ of male-derived MCs, or 1 × 10^7^ male-derived bone marrow (BM) cells. At 16 weeks after transplantation, BM, muscle, and spleen were sampled. (b) HSPCs in the MCs could home to the BM, muscle, and spleen of irradiated recipients. DNA samples were extracted, respectively, from the BM, muscle, and spleen, and male-derived cells were evaluated by PCR. Lane 1: female, Lanes 2–4: BM in control, MCs, and BM group, Lanes 5–7: muscle in control, MCs, and BM group, Lanes 8–10: spleen in control, MCs, and BM group, Lane 11: male, and Lane 12: marker. (c) Colony-forming unit (CFU) assay of BM cells derived from MCs transplantation recipients. Representative colonies were shown. Burst-forming unit-erythroid: BFU-E, colony-forming-unit-macrophage: CFU-M, colony-forming-unit-granulocyte, macrophage: GFU-GM, and colony-forming-unit-granulocyte, erythroid, macrophage, megakaryocyte: CFU-GEMM. (d) The percentage of individual Y-positive colonies in BM after MCs or BM transplantation was shown. The bars represented mean ± SD (*n* = 5); ^*^ *P* < 0.05 versus BM. (e) The survival numbers of mice in control, MCs, or BM transplantation.

To examine whether HSPCs in the MCs were capable of long-term hematopoietic reconstitution in vivo, 1 × 10^7^ MCs or BM cells derived from male mice were transplanted into irradiated syngenic female recipients. Y-chromosome PCR analysis was performed 16 weeks after transplantation (Figure 3(a)). The muscle, spleen, and BM of surviving recipients from groups 2 and 3 were demonstrated harboring a DNA fragment of Y-chromosome Sry region of donor mice, 239 bp in length, but no DNA fragments were detected in the BM, muscle, and spleen of any dead mice from three groups (Figure 3(b)), indicating that the transplanted MCs could home to the BM, muscle, and spleen of irradiated recipients, and survive in the irradiated mice. To confirm that the BM-homing male-derived cells derived from the MCs were functional HSPCs, we seeded BM cells from recipients on methylcellulose-based medium. The BM cells from surviving recipients gave rise to hematopoietic colonies (Figure 3(c)). To explore the frequency of Y-positive colonies in MCs or BM transplantation, we performed 20 individual hematopoietic colonies by Y-chromosome PCR analysis. The percentage of Y-positive colonies in MC transplantation was 92.78% ± 6.18% (*n* = 5, *P* = 0.001), whereas the percentage in BM transplantation was 81.25% ± 5.18% (*n* = 5) (Figure 3(d)), indicating that BM-homing MCs had hematopoietic activity and participated in hematopoiesis, and the number of these homing cells was more than that of BM-homing BM.

### 3.4. Rescue of Irradiated Mice

In this set of MCs transplantation experiments, all animals from group 1 (control group) died between days 9 and 13 after irradiation. In contrast, 9/15 mice from group 2 (MCs group) and 8/15 mice from group 3 (BM group) had survived for over 4 months (Figure 3(e)). This result showed that MCs transplantation was capable of generating long-term reconstitution in hematopoiesis. Moreover, MCs and BM were comparably efficient in reconstituting hematopoiesis.

### 3.5. HSPCs in the MCs Originated from the BM

**Figure 4:**
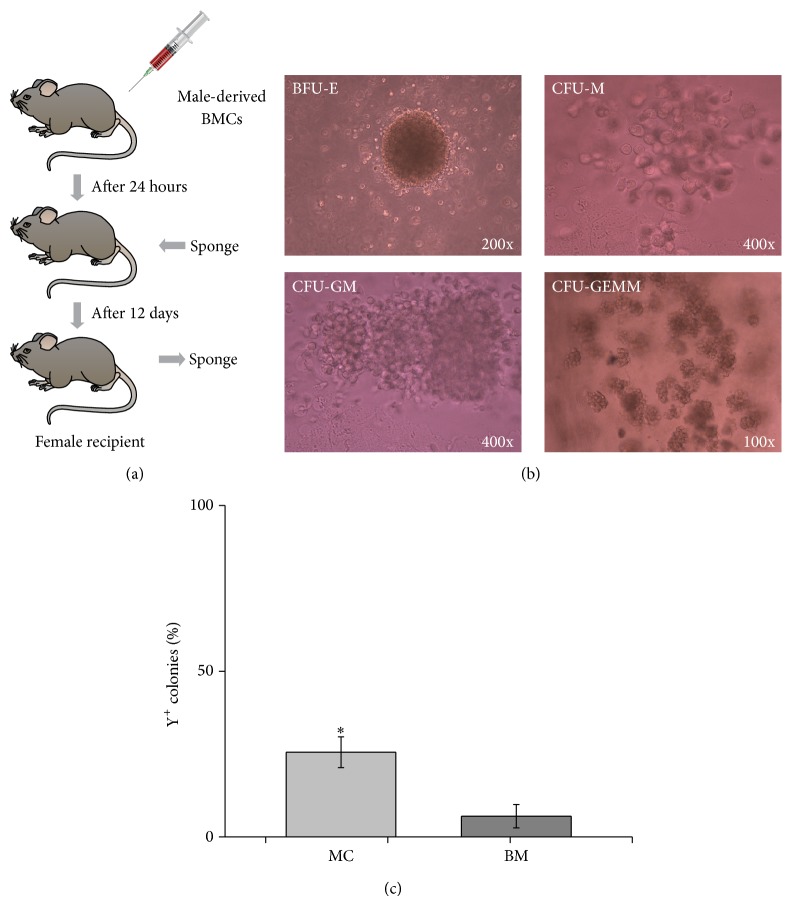
Hematopoietic stem and progenitor cells (HSPCs) in the migrating cells (MCs) originated in the bone marrow (BM). (a) Experimental scheme. 5 × 10^6^ BM cells were obtained from male mice and transplanted into female mice, and then sponges were implanted into the spatium intermusculare of female recipients at 24 hours after transplantation. The sponges were retrieved from recipients at 12 days after implantation and MCs were further analyzed. BM was obtained from female recipients as control. (b) Colony-forming unit (CFU) assay of MCs. Representative colonies were shown. BFU-E, colony-forming-unit-macrophage: CFU-M, colony-forming-unit-granulocyte, macrophage: GFU-GM, and colony-forming-unit-granulocyte, erythroid, macrophage, megakaryocyte: CFU-GEMM. (c) The percentage of individual Y-positive colonies in MCs was shown. The bars represented mean ± SD (*n* = 5); ^*^ *P* < 0.05 versus BM.

To elucidate whether HSPCs in the MCs originated from the BM, we performed BM transplantation (Figure 4(a)). The MCs from recipients could give rise to hematopoietic colonies (Figure 4(b)). To explore the frequency of Y-positive colonies in MCs, we performed 20 individual hematopoietic colonies by Y-chromosome PCR analysis. The percentage of Y-positive colonies in MCs was 25.56% ± 4.64% (*n* = 5), whereas it was 6.25% ± 3.54% in BM (*n* = 5, Figure 4(c)), indicating that HSPCs within the MCs could originate from the BM, and the approximation ratio of Y-positive colonies (MCs : BM = 4 : 1) was similar to that of hematopoietic colonies (see Figure 2(b) and Section 3.2).

### 3.6. Administration of G-CSF Increased HSPCs in the MCs

**Figure 5:**
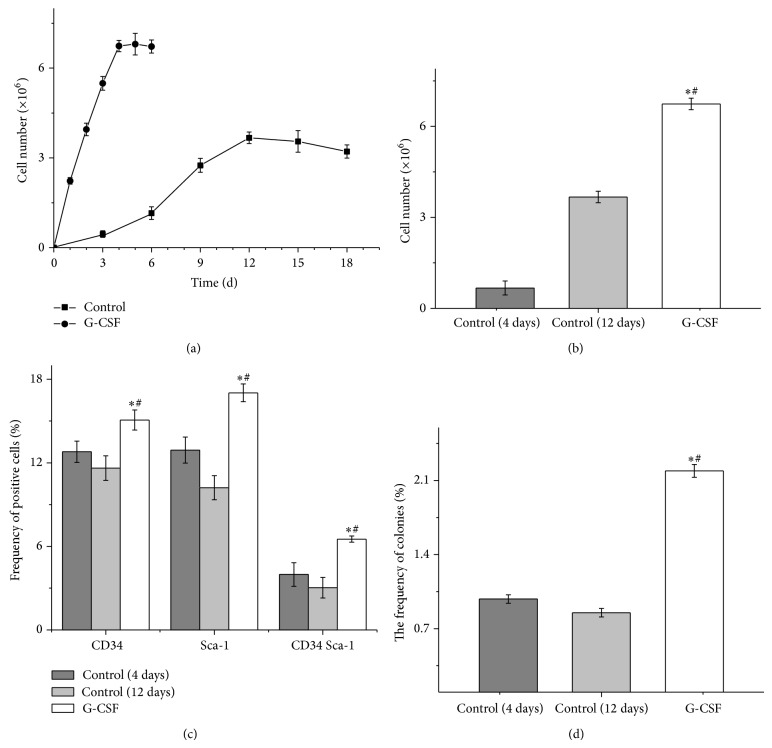
Granulocyte colony-stimulating factor (G-CSF) increased hematopoietic stem and progenitor cells (HSPCs) in the migrating cells (MCs). (a) Cell number in MCs after G-CSF administration. (b) G-CSF in the sponge reduced the time of implantation and increased the number of MCs. (c) G-CSF in the sponge significantly increased the frequency of CFU, CD34^+^, Sca-1^+^, and CD34^+^Sca-1^+^cells in the total MCs. (d) G-CSF in the sponge significantly increased the frequency of colonies in the total MCs. The bars represented mean ± SD (*n* = 5); ^*^ *P* < 0.05 versus control (4 days), ^#^ *P* < 0.05 versus control (12 days).

To increase HSPCs in the MCs, the sponge was soaked in G-CSF. After G-CSF administration, we found that MCs numbers were significantly increased and reached the maximum on the 4th day after implanting (Figure 5(a)). Therefore, the sponges were retrieved on the 4th day after implanting. G-CSF administration reduced the time of implantation and increased the number of MCs (Figure 5(b)). Moreover, G-CSF administration significantly increased the frequency of CFU, CD34^+^, Sca-1^+^, and CD34^+^Sca-1^+^ cells in the MCs compared with untreated group (all *P* < 0.05, Figures 5(c) and 5(d)). In addition, the frequency of each colony type in MCs treated with G-CSF was proportionally increased compared with untreated group; the proportion (BFU-E : GFU-GM : CFU-GEMM) of each colony type in MCs was similar (data not shown). These findings indicated that administration of G-CSF could not only greatly reduce the time of implantation, but also increase the number of MCs and frequency of HSPCs in the MCs.

## 4. Discussion

This study demonstrates for the first time that implanting biomaterials into the spatium intermusculare of mice can enrich HSPCs, which are capable of generating hematopoietic cells in an in vitro culture system as well as the in vivo long-term hematopoietic reconstruction. Moreover, the frequency is much higher than that in the BM. In addition, HSPCs in the MCs originate from the BM. Furthermore, the administration of G-CSF increases the number of HSPCs in the MCs. We suggest that HSPCs can be enriched by implanting biomaterial into spatium intermusculare. Currently, the most extensive method to harvest HSPCs is via apheresis. This method is almost noninvasive. Implantation of biomaterial to enrich HSPC is an invasive technique compared with apheresis. But this technique is not just as a method to enrich HSPCs. We have attempted to enrich autologous stem cells at the wound site by this technique. Through the biomaterials, autologous stem/progenitor cells could be enriched to the biomaterials at the wound site to facilitate the regeneration of lost tissues. It is very important for the limited regeneration capacity of adult tissue, such as heart and muscle tissue. Once encountering injury in big size, the activated stem cells are insufficient to repair injured tissue, despite the administration of G-CSF. Because the mobilized cells cannot be specifically recruited into the injury sites of heart and muscle tissue after the administration of G-CSF, implantation of biomaterial can increase the enriching capacity of biomaterial for autologous stem cells. We hypothesize that, due to the scaffold, the stem cells in circulation or from heart and muscle will stay on the scaffold, and gelatine membrane provides a scaffold for the cell proliferation and differentiation, which ultimately may promote the regeneration of injured tissue. The functional gelatine scaffold is transplanted into heart and muscle tissue as a patch to repair a surgical heart and muscle defect. Thus, the gelatine scaffolds could be an effective approach to promote tissue regeneration.

In this study, we successfully implant sponges into the spatium intermusculare of mice hind limbs and enrich a large number of MCs at the 12th day after implantation. In our previous study, the sponge was weighed, and the MCs number was counted successively at the 3rd, 6th, 9th, 12th,, and 18th days after implantation. The results showed that the number of MCs was significantly increased and reached the maximum after 12 days but sponge degraded after 12 days. We therefore suggest that the 12th day after implanting is a suitable time to retrieve the sponge.

A unique marker of the more primitive components (stem cells) in hematopoiesis remains to be identified. CD34 and Sca-1 are both used as phenotypic markers of relatively primitive hematopoietic progenitors [21, 22]. Colony assays are also used as an in vitro functional qualitation and quantitation of hematopoietic progenitors [12]. Our results demonstrate that MCs have typical HSPCs surface phenotypes CD34^+^ and Sca-1^+^, which form hematopoietic colonies, such as BFU-E-, CFU-M-, CFU-GM-, and CFU-GEMM-like HSPCs in the BM. Thus, MCs evidently contain HSPCs. These results lead us to investigate whether transplanted MCs are able to home to the BM and reconstitute long-term hematopoiesis in vivo. When HSPCs derived from BM are transplanted via the circulatory system, they imperatively home to the BM for proper proliferation and maintenance [20, 23–25]. This homing capacity is one of the instinct properties of HSPCs [8, 20]. BM ablated by irradiation constitutes a homing site for transplanted HSPCs [8, 20, 26]. Our results show that transplanted MCs, like transplanted BM cells, are found in the BM, muscle, and spleen, which is consistent with the recent report [27]. This characteristic localization of MCs in the BM may reflect a successful lodge on the part of MCs in the hematopoietic niche as definite HSPCs [20]. Thus, the BM homing of MCs may represent a reliable cue for the in vivo functionality of HSPCs in the MCs. Our results also reveal that HSPCs in the MCs successfully engraft into the BM and achieve long-term (16 weeks) reconstitution of hematopoietic cells in irradiated recipients. This phenomenon is in marked contrast to transiently reconstituted hematopoietic cells, which are usually undetectable by 8 to 10 weeks after transplantation [28]. Moreover, BM-homing MCs have hematopoietic activity and MCs and BM are comparably efficient in reconstituting hematopoiesis. These results are compelling evidence that the MCs contain functional HSPCs.

In addition, we also use the same method as the previous study (BM transplantation) to elucidate the origin of HSPC in MCs. Our results demonstrate that HSPCs in the MCs originate from the BM. The HSPCs in the BM, like MSCs, constantly move from the BM to systemic circulation and home to the BM and extramedullary tissues by an identified mechanism. Supporting this concept, several extramedullary tissues are identified as HSPC-containing organs, including the liver [9], spleen [10], muscle, [11], adipose tissue [12], and thoracic duct [13]. Stem cells including MSCs and HSPCs have been also reported to be able to migrate and home to sites of inflammation following tissue injury [29]. In this study, surgical procedure of implanting sponges do not cause traumatic bleeding, so we speculate the similar possible mechanism with the previous study, including (1) hypoxic environment [30]; (2) characteristic vascular system [19, 31]; (3) 3D porous structure [32]; and (4) inflammatory response. Based on these possibilities, we propose that HSPCs and MSCs derived from BM could migrate into biomaterial and maintain their properties and function properly. Further studies are needed to determine what circumstances can affect homing and the mobilization of HSPCs within the MCs.

Because HSPCs in the MCs originate from the BM, we investigate whether HSPCs in the MCs increase by enhanced mobilization. Circulation is the reservation of autologous stem cells, including HSPCs, MSCs, and endothelial progenitor cells. Previous studies have shown that G-CSF could enhance the stem cell proliferation and mobilization, but not the maturation rate, of murine myeloid cells [33]. In our study, whether G-CSF enhances the maturation of neutrophilic granulocytes or just accelerates the mobilization of mature and maturing granulocytes from blood to MCs, or both, is not clear. We will answer this question in our further research. Our results indicate that administration of G-CSF could not only greatly reduce the time of implantation, but also greatly increase the MCs number and frequency of HSPCs in the MCs.

## 5. Conclusions

According to our present data, the estimated amount of HSPCs in total MCs would be equivalent to approximately 4–6-fold of that of HSPCs in the total BM. Moreover, our results suggest that more HSPCs could be obtained from the MCs if we use G-CSF. These data provide compelling evidence that HSPCs can be enriched by implanting biomaterial into spatium intermusculare. Implantation of biomaterial may be seen as the first step to a proof of their applicability to clinical practice in enriching HSPCs.

## Abbreviations

3D: Three-dimensional
HSPCs: Hematopoietic stem and progenitor cells
MSCs: Mesenchymal stem cells
PB: Peripheral blood
BM: Bone marrow
MCs: Migrating cells
PBS: Phosphate-buffered saline
FBS: Fetal bovine serum
Sca-1: Stem cell antigen
FITC: Fluorescent isothiocyanate
PE: Phycoerythrin
CFU: Colony-forming unit
BFU-E: Burst-forming unit-erythroid
CFU-M: Colony-forming-unit-macrophage
CFU-GM: Colony-forming-unit-granulocyte, macrophage
CFU-GEMM: Colony-forming-unit-granulocyte, erythroid, macrophage, megakaryocyte
PCR: Polymerase chain reaction
dNTP: Deoxyribonucleotide triphosphates
G-CSF: Granulocyte colony-stimulating factor
SD: Standard deviation
PLA: Polylactic acid.
